# Hepatitis B Vaccination in Psychiatric patients in Morocco

**DOI:** 10.1192/j.eurpsy.2023.1586

**Published:** 2023-07-19

**Authors:** B. Zineb, T. Aicha, K. Imane, L. Fouad, O. Abderrazak

**Affiliations:** 1 Ar-razi psychiatric hospital, Faculty of Medecine and Pharmacy; 2 Ar-razi Psychiatric hospital, Rabat, Morocco

## Abstract

**Introduction:**

Severe mental illnesses such as schizophrenia, bipolar disorder and depressive disorder are common worldwide and often have a chronic course.

Due to psychiatric ( for example : substance use disorders ) and somatic ( for example : obesity, cardiovascular disease, diabetes ) comorbidities, mortality is higher in these patients than in the general population.Viral diseases are, in addition to cardiovascular diseases and metabolic alterations, among the most common somatic comorbidities in people with severe mental illness.

**Objectives:**

The objective of our work was to study the prevalence of vaccination against the viral hepatitis B virus in these patients.

**Methods:**

For this purpose, we conducted a cross-sectional study of 200 patients hospitalized in the emergency department of our training center. First, we collected sociodemographic and clinical data on patients admitted to psychiatric emergencies: sex, age, diagnosis, duration of evolution, history (medical and surgical, psychiatric, suicide attempts, problematic substance use, previous incarceration). In a second step, we tested for anti-HBS antibodies.

**Results:**

the majority of our patients were male, the first diagnosis was schizophrenia.

92% of our patients had a substance use disorder, mainly tobacco, followed by cannabis and then alcohol.

Several patients reported having unprotected sex.

58% of patients were vaccinated against hepatitis B, these patients were young for the most part. the majority of our patients were male, the first diagnosis was schizophrenia.92% of our patients had a substance use disorder, mainly tobacco, followed by cannabis and then alcohol.Several patients reported having unprotected sex.58% of patients were vaccinated against hepatitis B, these patients were young for the most part.

**Conclusions: Disclosure of Interest:**

None Declared

